# Using a Representative Sample of Elementary School Students to Determine the Statewide Prevalence of Childhood Overweight or Obesity in Utah

**Published:** 2009-09-15

**Authors:** Karen Nellist, Karen Coats, Mike Friedrichs

**Affiliations:** Utah Department of Health, Bureau of Health Promotion; Utah Department of Health, Salt Lake City, Utah; Utah Department of Health, Salt Lake City, Utah

## Abstract

Utah's Height and Weight Measurement Project was conducted with elementary school students periodically from 2002 to 2008. The 2002 pilot project was performed to establish variability rates between schools and within schools. It allowed us to accurately determine both the sample size and the number of schools that were required to calculate a reliable statewide estimate based on a random sample of schools and to establish sentinel grades. The sentinel grades identified were grades 1, 3, and 5. Use of randomly selected classes in the sentinel grades resulted in decreased sample size and less school disruption while maintaining sufficient precision. Standardized, calibrated equipment was purchased and given to school nurses for safekeeping. Lessons learned included establishing strong relationships with partners, especially school nurses, and obtaining support from upper management at the schools, school districts, and the Utah Department of Health. A significant difference in participation rates and obesity rates at the individual school level was observed depending on parental consent type; active consent was associated with lower student participation rates and lower observed obesity rates. Data were presented to both participating and nonparticipating schools, school nurses, district superintendents, and principals. For surveillance purposes, sampling is an efficient, cost-effective way to estimate childhood overweight and obesity rates.

## Background

Overweight and obesity in children, young adults, and adults continues to be a problem nationwide. To quantify the extent of this problem and to monitor response to interventions, a statewide prevalence rate must be established. Data for self-reported statewide height and weight are available for adults though the Behavioral Risk Factor Surveillance System (BRFSS) telephone survey, and similar data are available for high school students through the written Youth Risk Behavior Surveillance System (YRBSS). Both the BRFSS and the YRBSS are federally funded surveys designed to obtain state- and national-level data. Height and weight data (directly measured) are available for children through the federally funded National Health and Nutrition Examination Survey (NHANES), though this survey is designed to obtain only national rates. Thus, to obtain statewide overweight and obesity prevalence in elementary school students, the state would have to conduct its own surveillance.

A review of how different states have approached the task of measuring the prevalence of childhood obesity and overweight and the related issues was recently conducted (1). States have used various methods to obtain statewide childhood obesity and overweight prevalence rates. Some states have obtained height and weight data from routine statewide fitness evaluations; others have weighed and measured every child (ie, performed a census). To control cost and minimize school disruption, the Utah Department of Health (UDOH) estimated childhood overweight and obesity on the basis of a representative random sample of elementary school students. The project was successful because of solid relationships with partners, especially school nurses; strong support in UDOH and local health departments; and buy-in from the school districts. Utah's Height and Weight Measurement Project has weighed and measured Utah elementary school students periodically from 2002 through 2008.

### Why data on childhood overweight and obesity statewide prevalence are needed

State-level childhood overweight and obesity data are essential to establishing state-level needs and plans to decrease childhood overweight and obesity. State-specific childhood body mass index (BMI) data can help state legislators understand the health implications of obesity. Additionally, the data can be presented to a cross-section of people and businesses involved in children's lives to form a compelling case for targeted interventions in the public and private sectors. The data can be used to establish funding priorities and to evaluate intervention efforts.

Data from the 2002 pilot project were presented at the first Utah Obesity Summit in 2005, which led to the establishment of a nonprofit organization, the 501[c]3 Utah Partnership for Healthy Weight (UPHW), comprising representatives from the public and private sectors. UPHW uses these data to encourage people and organizations to participate in prevention and intervention programs. The 2006 data were presented at various meetings, including the Rocco C. and Marion S. Sicilian Forum, which is sponsored by the University of Utah, the Utah School Nurses Association (USNA) 2007 Fall Conference, and the Utah state legislature (2,3).

These data helped educate students and members of the public who attended the forum and proved that Utah needs to address childhood obesity. The presentation at USNA showed the nurses how the collected data can measure the success or failure of interventions. In 2002, a school district used the pilot project data to obtain a small physical activity education grant. The data were used to establish a need in federal grant applications. The Centers for Disease Control and Prevention awarded Utah a 5-year Physical Activity, Nutrition, and Obesity (PANO) grant in 2008 after a competitive review process. A substantial part of the PANO grant application was dedicated to establishing evaluation criteria to measure intervention effectiveness. The evaluation plan, including statewide childhood overweight and obesity measurements over time, strengthened the application.

### Legal issues

Unlike in other states, in Utah, height, weight, and BMI reports were not given to the student or to parents. Early in the program, individual school reports were sent to participating schools so that they could compare their obesity rate with the state obesity rate. We did not send BMI report cards home to the parents because this program was designed for surveillance.

The issue of school and parental consent had to be addressed. In Utah, the school district superintendent was presented with information about the project, and approval to perform the study was requested. After district approval was obtained, the school district nurses spoke with school principals to determine if passive parental consent was acceptable or if active parental consent was required. Our preference was passive consent. Data from the first year of this surveillance study showed that passive consent resulted in higher participation rates, which in turn yielded a higher prevalence of overweight and obesity.

In compliance with Health Insurance Portability and Accountability Act (HIPAA) regulations, no student names were collected; only birth date, sex, height, and weight were collected. Information about who performed the height and weight measurements is always collected to expedite any necessary data cleaning. The data were entered into a database on a secured server that was accessible with a protected password.

### Need for a pilot study

The pilot study was performed in 2002 to establish variability rates between and within schools. Obtaining these variability rates allowed us to more accurately determine both the sample size of students and the number of schools that were required to determine a reliable statewide estimate. During the pilot study we weighed and measured children in 27 schools. Urban and nonurban schools were included in the convenience sample. A group of 3 schools was randomly selected in 9 urban and 9 rural areas statewide; the school nurse selected 1 of the 3 schools in each set at his or her convenience. During the pilot study we measured all children in kindergarten through grade 8 in the selected school and were able to determine that the results for grades 1, 3, and 5 sufficiently represented all the students in kindergarten through grade 6. This determination allowed us to reduce the number of students sampled, control costs, and still determine a reliable statewide estimate. The within- and between-school variability rates were measured. The measured variability within schools was smaller than the measured variability between schools. These results taught us that we could weigh and measure fewer children in each school but would have to include more schools in the sample.

## Relationships With Partners, Especially School Nurses

A written project proposal was generated to share with internal and external UDOH partners. External partners included school nurses, school district superintendents, and school principals. Representatives from the school nurses and some internal UDOH partners reviewed the project proposal before it was finalized to increase stakeholder input.

School district approval was obtained as follows. Approximately 1 year before the actual weighing and measuring occurred, UDOH sent a letter, the project proposal, and an approval signature sheet to the district superintendent for each randomly selected school. Most superintendents faxed the approval signature sheet to UDOH without requesting additional information or applications. Some school districts required UDOH to submit an application or wanted to speak with the responsible epidemiologist. All randomly selected school districts gave their approval to conduct the study.

Support from the participating school nurses was established as follows. The UDOH, through a school nurse liaison, determined the best way to communicate with school nurses. A booth was secured at the spring Utah school nurses conference, and the school nurses from the participating schools were invited to visit the booth and learn more about the project. School nurses from participating schools who did not attend the conference were mailed relevant materials.

An e-mail group was created to communicate with school nurses from participating schools. Feedback on the methods to be used was solicited from the school nurses, and their responses, in some instances, resulted in a change in methods. Specifically, the school nurses expressed a concern about the time it would take to weigh all first-, third-, and fifth-grade students in the school. In response the method was changed so that 1 class from each of the 3 grades was randomly selected, and all of the students in that class were weighed and measured. An e-mail survey was conducted among school nurses to determine the most convenient time of year to conduct the study; January 2 to March 31 was chosen. Training was offered as a PowerPoint presentation on compact disc that was mailed to participating school nurses. UDOH offered help finding volunteers to help weigh and measure if they were needed. UDOH communicated routinely with participating school nurses via e-mail for the duration of the project and followed up by telephone if necessary; 6 e-mails were sent to the participating school nurses during the school year.

The support of school administrators was obtained as follows. A letter was developed for the principals of each selected school; UDOH gave the school nurses the option of delivering the letter to capitalize on established relationships. If the participating school nurses elected not to deliver the letter to their principal, UDOH mailed the letter and followed up with a telephone call to ensure that approval was obtained. If the principal declined school participation, a substitute school was selected from the second list of randomly selected schools and the process of obtaining principal support was repeated.

Consent to weigh and measure students was obtained as follows. Though the preference was for passive consent, some school districts required active consent. In all cases a consent form was sent home with the student before any weighing and measuring was done. Consent forms were created by UDOH in both Spanish and English, and the participating school nurse decided whether to use the UDOH form or an alternative form.

## Equipment, Training, and Data Collection

Research into the type and make of scales as well as portable ways to measure children's height resulted in the selection of the Tanita BWB 800 AS digital scale (Tanita Corporation of America, Inc, Arlington Heights, Illinois) and the Handi-Stat Portable Stadiometer with a metal tape measure. Information on standardized methods for weighing and measuring children was reviewed, and because no CDC protocol was available in 2001, a standard method was developed (4).

For the first year of the project (2006), all required equipment was purchased by UDOH and mailed to participating schools or directly to the participating school nurses, depending on recommendations from the school nurse. After weighing and measuring were completed, instructions on how to correctly store all equipment were sent to the school nurses and school principals. Before the start of the second weighing and measuring period (2008), the location of all equipment was determined, and 86% of the scales were checked for accuracy to 72 pounds (59 scales); no scale required calibration.

Included with the mailing of the compact disc training presentation were a 1-page height and weight measurement methods sheet, a school data form, and a postage-paid return envelope. An updated training CD was included in 2008. Volunteers who performed the weighing and measuring without a school nurse present received hour-long, in-person trainings from UDOH or the local health department.

The data collection form included the school name and identification number; teacher name and grade; scale number and information on who performed what measurement; and individual student data (birth date, sex, height, and weight). The school data form included the number of students in the entire school, number of students in first, third, and fifth grades, and number of students in the randomly selected classes. Participating school nurses were asked to retain a copy of the data collection forms at their school and send the originals to UDOH. On receipt of the data collection forms, UDOH made copies, reviewed the data for inconsistencies, and contacted the school nurse if errors or omissions were found. Data were entered into a database.

## Statistical Methods

The number of schools needed (*m*) was identified by using a sample size calculation for a simple 2-stage cluster sampling (5):

**Equation 1.** Sample Size Calculation for the Utah Height and Weight Surveillance Study

m=(σ1x2X ¯ 2)×(MM−1)+(1n ¯ )×(σ2x2X ¯ 2)×(N ¯ −n ¯ N ¯ −1)ε2Z1−(α÷2)2+σ1x2X ¯ 2(M−1)

Where:

σ1x2=variance among schools

 

σ2x2=variance among units in a school

 

n ¯ =sample size in each school

 

σ1x2=ΣM(Xi−X¯)2M

 

σ2x2=ΣMΣN(Xij−Xi¯)2N

The schools were randomized, and a class in each school was randomly selected. The randomization was performed by using SAS version 9.1.3, service pack 4 (SAS Institute, Inc, Cary, North Carolina). Though the intent was to collect data from 74 schools, only 69 schools chose to participate. The schools that were identified during the first of 3 measuring periods (2006) were used for the second measuring period (2008) and will be used again during the third measuring period (2010). If a school closed or no longer wanted to participate in the project, the school was replaced with a similar school in terms of location and district (zip code) in an attempt to minimize variability. The intent when selecting replacement schools was to follow the children from a school that had closed to the new school, to measure children exposed to the same social and environmental pressures, or both. The 69 schools represented both urban and rural areas.

Student data were categorized by BMI (ideal weight, overweight, and obese) on the basis of standardized growth curves and classifications established by CDC. The growth charts are available at the National Center for Health Statistics Web site (http://www.cdc.gov/GrowthCharts/). SAS code that executes this classification is available free from CDC (6). *The z* scores from growth charts were inserted into the database.

Weighting of the data from participating schools in order to extrapolate to the entire state took into consideration the probability that the school was selected and the probability that a certain child was selected given that the school was selected. The formula used to determine the weight for each child is shown in Equation 2.

**Equation 2.** Weighting Formula for the Utah Height and Weight Surveillance Study

Probability a student was selected=student countnumber of classes per grade×class count

Because bias could not be measured, variance was used to determine whether the resulting statewide estimate was precise by comparing the data-derived variance with the variance determined during the pilot project. In our case, these variances were similar.

## Sharing Data With Partners

After the first year of the study (2006), a report was developed and sent to participating school nurses, all school districts, and all elementary schools in 2007. The report included national and state data, information with references about how overweight or obesity affects children, and recommendations for actions that schools could take to support a healthy weight in students. UDOH presented the results of the study at the 2007 fall Utah school nurses conference, and the report was distributed at the presentation  (7,8).

## Lessons Learned

As expected, the response rate for the schools with active consent was less than the response rate for schools with passive consent (74.4% vs 90.7%). Calculation of overweight and obesity rates by consent type (passive vs active) showed a significant difference in the estimates of obesity by consent type. Obesity rates for girls in active-consent schools were significantly lower than those for girls in passive-consent schools (*P* < .05); for boys this difference approached statistical significance (*P* = .06). Statistical adjustment for active consent showed a 1% increase in the overall obesity rate. In other words, active consent may have resulted in an underestimation of the obesity rate. Passive consent is preferable to active consent.

Upper management support was critical to success. Support within UDOH resulted in more employees being assigned to help the lead coordinator with paperwork and follow-up telephone calls, and support from the school district superintendent allowed the school nurses to weigh and measure students more easily. The 2006 data were summarized in a 4-page handout that was sent to the schools both electronically and by mail. The amount of paperwork and extent of coordination efforts were manageable because of the limited number of schools.

Identification of grades 1, 3, and 5 as being representative of all elementary schools allowed us to decrease the sample size and still obtain an accurate state-level estimate of the prevalence of childhood obesity. In fact, the grade 3 rate was not different from the state rate (calculated by combining data from grades 1, 3, and 5).

Being able to determine the statewide childhood overweight and obesity prevalence rates has been useful. The rates have been included in statewide reports, grant applications, information for the state legislature, and newspaper articles. Additionally, the data have been included in presentations to various audiences including a statewide coalition for healthy weight, a statewide meeting for cities and towns, a forum on obesity at the University of Utah, a statewide school nurses conference, and peer-reviewed journal articles (4). Knowing the statewide childhood obesity rate helps to explain the long-term consequences of obesity from childhood to adulthood. In our case, fifth-grade boys were identified as a key group for intervention because the obesity rate for boys dramatically increased from third to fifth grade.

Each school was responsible for its equipment. Because the number of schools was limited and the same schools participate every time, we limited the number of scales and equipment purchased. We purchased 80 scales and shipped 69 for a one-time charge of $48,000. The remaining scales were stored as possible replacements for damaged or lost equipment. One secretary was able to track all data received and make follow-up calls for data clarification, resulting in minimal transcription errors and more complete data. The secretary was able to perform these duties in addition to her normal workload. Additionally, only 20% of an employee's time was required to coordinate the study. Database entry costs were contained to data from 4,000 students; minimal analysis time was required.
